# Retraction: DEPDC1 promotes cell proliferation and suppresses sensitivity to chemotherapy in human hepatocellular carcinoma

**DOI:** 10.1042/BSR-2019-0946_RET

**Published:** 2024-07-12

**Keywords:** cell proliferation, chemotherapy resistance, DEPDC1, hepatocellular carcinoma, JNK signaling pathway

This article is being retracted from Bioscience Reports following receipt of a notification from a reader, alerting the Editorial Office to Figure 3C pLKO.1 panel which seems to appear as Figure 4D 5-FU pLKO.1 panel from Xu et al, 2020 (DOI: 10.1111/jcmm.15070) with contrast adjustment and addition of a colour channel.

The authors were contacted with regards to the concerns raised, and state that the experiments in this paper were conducted by an external technical company, which was not declared in the article or during the submission process. The authors were asked to provide the raw data for the figures, however the images provided during the investigation differed from those in the published paper. The authors requested additional time to recover the original data, however given the concern surrounding the scientific validity of this paper, the Editorial Board stands by the decision to retract. The authors disagree to the retraction.

